# Vitamin B12 deficiency among patients with diabetes mellitus: is routine screening and supplementation justified?

**DOI:** 10.1186/2251-6581-12-17

**Published:** 2013-05-07

**Authors:** Davis Kibirige, Raymond Mwebaze

**Affiliations:** 1 Department of Medicine, St. Raphael of St. Francis hospital Nsambya, P.O.BOX 7146, Kampala, Uganda; 2 Diabetes and endocrine clinic, St. Raphael of St. Francis hospital Nsambya, Kampala, Uganda; 3 Mother Kevin Postgraduate Medical School, Uganda Martyrs University Nkozi, Nkozi, Uganda

**Keywords:** Vitamin B12 deficiency, Diabetes mellitus, Screening, Supplementation

## Abstract

Vitamin B12 is an essential micronutrient required for optimal hemopoetic, neuro-cognitive and cardiovascular function. Biochemical and clinical vitamin B12 deficiency has been demonstrated to be highly prevalent among patients with type 1 and type 2 diabetes mellitus. It presents with diverse clinical manifestations ranging from impaired memory, dementia, delirium, peripheral neuropathy, sub acute combined degeneration of the spinal cord, megaloblastic anemia and pancytopenia. This review article offers a current perspective on the physiological roles of vitamin B12, proposed pathophysiological mechanisms of vitamin B12 deficiency, screening for vitamin B12 deficiency and vitamin B12 supplementation among patients with diabetes mellitus.

## Introduction

Vitamin B12 or cobalamin is a water soluble vitamin that plays a very fundamental role in DNA synthesis, optimal haemopoesis and neurological function. The clinical picture of vitamin B12 deficiency hence, is predominantly of features of haematological and neuro-cognitive dysfunction [1].

This review will mainly discuss the physiological roles of vitamin B12, the varied pathophysiological mechanisms of vitamin B12 deficiency among patients with type 1 and 2 diabetes mellitus (DM) and perspectives on screening for vitamin B12 deficiency and supplementation of vitamin B12 among diabetic patients.

### Absorption of vitamin B12

The principal source of vitamin B12 is animal proteins. The preliminary step in the metabolism of vitamin B12 involves its release from animal sources, a process mediated by the action of pepsin and gastric acid. After the release, dietary vitamin B12 binds to the R-protein secreted by the salivary glands. In the duodenum, in the presence of an alkaline medium and pancreatic proteases, the R- protein is hydrolysed to release vitamin B12 which later binds with the intrinsic factor (IF) secreted by the gastric parietal cells.

The vitamin B12 –IF complex is highly resistant to proteolytic degradation. The complex attaches at its specific receptors on the mucosa of the terminal ileum, a site where its absorption occurs. This stage of vitamin B12 absorption is calcium mediated.

The intracellular vitamin B12 is released following IF degradation. This free vitamin B12 attaches to another protein carrier, transcobalamin –II (TC-II) and is later released into the circulation. This vitamin B12 – TC-II complex, also referred to as holo TC-II is then actively taken up by the liver, bone marrow and other vital body cells. The liver serves as the principal storage site of up to 90% of the body’s total vitamin B12 [1,2].

A disruption in any of the described steps above will result into clinical or biochemical vitamin B12 deficiency. This includes insufficient dietary intake especially among alcoholics and vegetarians and malabsorption due to several conditions like chronic atrophic gastritis mainly in the elderly, pernicious anemia, celiac disease, chronic pancreatitis and drugs like metformin and proton pump inhibitors (PPIs).

### Physiological roles of vitamin B12

Vitamin B12 exerts its physiological effects through mediating two principal enzymatic pathways i.e. the methylation process of homocysteine to methionine and the conversion of methylmalonyl coenzyme A (CoA) to succinyl-CoA. Vitamin B12 as a co-factor facilitates the methylation of homocysteine to methionine which is later activated into S-adenosyl-methionine that donates its methyl group to methyl acceptors such as myelin, neurotransmitters and membrane phospholipids.

Metabolically significant vitamin B12 deficiency hence will result in disruption of the methylation process and accumulation of intracellular and serum homocysteine. Hyperhomocysteinemia has been shown to have potentially toxic effects on neurones and the vascular endothelium. This reaction is also essential in the conversion of dietary folate (methyl-tetrahydrofolate) to its active metabolic form, tetrahydrofolate. In another essential enzymatic pathway, vitamin B12 as a co-factor mediates the conversion of methylmalonyl coenzyme A (CoA) to succinyl-CoA. In the presence of vitamin B12 deficiency, this conversion pathway is diminished and an increase in the serum methylmalonic acid (MMA) ensues. This is followed by defective fatty acid synthesis of the neuronal membranes [3]. Vitamin B12 is also essential in the synthesis of monoamines or neurotransmitters like serotonin and dopamine [4]. This synthesis is impaired with vitamin B12 deficiency.

All the above collectively explain the resultant neuro-cognitive or psychiatric manifestations that accompany vitamin B12 deficiency. Axonal demyelination, degeneration and later death are the hallmark of vitamin B12 deficiency induced neuronal damage that manifests as severe peripheral or autonomic neuropathy, sub acute combined degeneration of the spinal cord, delirium and dementia [3,5]. Compelling evidence demonstrates that hyperhomocysteinemia is also associated with an increased risk of cardiovascular events due to its cellular and vasculo-toxic effects [6-8].

Vitamin B12 is an essential micronutrient required in DNA synthesis, cellular repair and normal haemopoesis together with other micronutrients like folate and iron. Vitamin B12 deficiency is classically associated with overt haematological findings like macrocytic red blood cells (mean cell volume [MCV]> 100 fl) with/without anaemia, ovalocytes, hyper segmented white blood cells (i.e. >5% of neutrophils with ≥5 lobes) and pancytopenia [9]. Due to defective cell repair processes, atrophic glossitis, stomatitis and malabsorption due to villi atrophy and mucositis are also common.

### Vitamin B12 deficiency among patients with type 2 diabetes mellitus and the general population: a comparative review

Several cross sectional studies [10-12] and case reports [13-15] have documented an increased frequency of vitamin B12 deficiency among type 2 DM (T2DM) patients. Metformin use has been unequivocally demonstrated as the prime factor associated with vitamin B12 deficiency among patients with T2DM [16-19]. Studies assessing type 2 diabetic patients on metformin have reported the prevalence of vitamin B12 deficiency to range from 5.8% to 33% [10,19,20]. This wide variation in the reported prevalence could probably be explained by the varied study definitions of vitamin B12 deficiency. In the cross sectional study by Pflipsen et al. on 203 outpatient type 2 diabetic patients at a large military primary care clinic in USA, definite vitamin B12 deficiency was defined as serum vitamin B12 concentrations of <100 pg/ml or elevated serum methylmalonic acid of >243 nmol/L or homocysteine concentrations of >11.9 nmol/L if serum vitamin B12 concentrations were between 100 to 350 pg/mL [10]. Reinstatler et al. in the National Health and Nutrition Examination Survey of 1999–2006 in the USA defined definite and borderline biochemical vitamin B12 deficiency as serum vitamin B12 concentrations of ≤148 pmol/l and >148-221 pmol/l respectively [19]. In one cross sectional study that documented a high prevalence of vitamin B12 deficiency of 33% among adult patients with T2DM by Qureshi et al., vitamin B12 deficiency was defined as serum vitamin B12 concentrations <150 pg/ml [20]. However, patients enrolled in this study were those who were on high dose (>2 g/day) and long-term (4 years) metformin treatment, both clinical factors known to be associated with vitamin B12 deficiency [17,18].

Due to the diverse definitions of vitamin B12 deficiency used in most studies and the cultural and religious beliefs in different regions of the world, comparison of the prevalence of vitamin B12 deficiency among T2DM patients and healthy general populations is difficult. In one population based study among 1048 elderly Finnish subjects aged 65–100 years, the total prevalence of definite vitamin B12 deficiency was 12.1% [21]. Previously diagnosed vitamin B12 deficiency was reported among 2.6% of the participants. Vitamin B12 replacement therapy was documented among only 2.6% of the participants. In this study, vitamin B12 deficiency was defined as total serum vitamin B12 concentrations *<*150 pmol/l or total serum vitamin B12of 150–250 pmol/l and holotranscobalamin ≤37 pmol/l and homocysteine ≥15 μmol/l.

In India, a country with a large proportion of vegetarians due to cultural and religious beliefs, very high prevalence of vitamin B12 deficiency among the general population has been reported. In one study by Yajnik et al. to determine the frequency of vitamin B12 deficiency and hyperhomocysteinemia among 441 healthy middle aged Indian men, vitamin B12 deficiency as defined by vitamin B12 concentrations <150 pmol/L was reported among 67% of the study participants [22]. Vegetarian diet was the sole significant factor associated with low vitamin B12 levels in this study on multivariate analysis (OR 4.4 95% CI 2.1-9.3).

In another cross sectional study among 175 healthy elderly Indian subjects aged >60 years, vitamin B12 deficiency also defined as vitamin B12 concentrations <150 pmol/L was reported among 16% of the study participants [23]. Elevated serum MMA concentrations which are a more sensitive indicator of vitamin B12 deficiency were documented among 55% of the participants.

### Metformin induced vitamin B12 deficiency among patients with T2DM

In the absence of contraindications like renal and hepatic dysfunction, recent guidelines advocate for the use of metformin as the first line glucose lowering agent concurrently with life style modification approaches [24,25]. Despite its very superior glycemic lowering effect, metformin has for long been shown to decrease vitamin B12 levels. In one early randomised controlled trial by DeFronzo et al., metformin decreased the serum vitamin B12 levels by 22% and 29% compared to placebo and glyburide respectively [26]. This side effect of metformin has been demonstrated again in several ensuing cross sectional studies [10-12], case reports [13-15] and randomised controlled trials [16,17].

The risk of developing metformin associated vitamin B12 deficiency is greatly influenced by increasing age, metformin dose and duration of use [17,18]. In a nested case control study performed among 155 adult Chinese DM patients on metformin and 310 controls, every 1 g/day increase in the metformin dose conferred an odds ratio of 2.9 (95% confidence interval, 2.15-3.87) for developing vitamin B12 deficiency. Among patients using metformin for ≥ 3 years, the adjusted odds ratio was 2.4 (95% confidence interval, 1.46-3.91) compared with those who had received metformin for ≤ 3 years [18].

Decrease in vitamin B12 absorption and levels following metformin use typically starts as early as the 4^th^ month [27]. Clinically overt features of vitamin B12 deficiency manifest by 5–10 years owing to the large body stores in the liver mainly that are not quickly depleted [28].

The proposed mechanisms to explain metformin induced vitamin B12 deficiency among patients with T2DM include: alterations in small bowel motility which stimulates bacterial overgrowth and consequential vitamin B12 deficiency, competitive inhibition or inactivation of vitamin B12 absorption, alterations in intrinsic factor (IF) levels and interaction with the cubulin endocytic receptor [28]. Metformin has also been shown to inhibit the calcium dependent absorption of the vitamin B12-IF complex at the terminal ileum. This inhibitory effect is reversed with calcium supplementation [29].

### Vitamin B12 deficiency among patients with type 1 diabetes mellitus

Type 1 DM (T1DM) is an auto immune condition that results from auto immune destruction of insulin secreting beta cells of the pancreas. It is invariably associated with other organ and non organ specific auto immune and endocrine conditions leading to development of autoimmune polyglandular syndromes [30].

Pernicious anemia resulting from chronic autoimmune gastritis is highly frequent among patients with T1DM. Chronic autoimmune gastritis and pernicious anemia occurs in about 2% and up to 1% of the general population respectively. Among patients with T1DM, the prevalence is increased by 3 to 5 folds [31].

Vitamin B12 deficiency due to pernicious anemia occurs frequently among patients with T1DM. In one cross sectional study done in South India among 90 patients with T1DM, low vitamin B12 levels were noted among 45.5% of the study subjects as defined by the manufactures’ cut off point of <180 pg/ml and among 54% using the published cut off point of <200 pg/ml [32]. No positive correlation was noted between low vitamin B12 levels and gender, age, duration of DM and level of glycemic control.

Patients with T1DM actively exhibit auto antibodies to intrinsic factor (AIF) type 1 and 2 [31] and parietal cell antibodies (PCA) [33,34] especially those with glutamate decarboxylase-65 (GAD-65) antibodies and HLA-DQA1*0501-B1*0301 haplotype [35].

The PCA inhibit secretion of intrinsic factor resulting into pernicious anemia, a condition which is 10 times more prevalent among type 1 diabetic patients than non diabetic patients. Type 1 AIF result into vitamin B12 deficiency by blocking the binding of vitamin B12 to IF. This prevents its transportation to its absorption site, the terminal ileum. These auto antibodies are found in 70% of patients with pernicious anemia [31].

Primary autoimmune hypothyroidism and celiac disease are frequent co morbidities among patients with T1DM [36-38] and directly affect vitamin B12 metabolism [39,40]. In one cross sectional study among 504 ambulatory T1DM patients in South Africa, the overall prevalence of co-existing auto immune hypothyroidism was 20.2%, especially among female patients (30.9% Vs 10.1%-males, p<0.0002). Celiac disease in this study cohort was reported in 3 (0.6%) patients [37].

Vitamin B12 deficiency among patients with autoimmune hypothyroidism could be explained by the presence of antibodies to the gastric parietal cells and intrinsic factor, reduced oral intake, dyserythropoesis due to thyroid hormone deficiency and defective absorption due to reduced bowel motility, bowel wall oedema and bacterial overgrowth [40].

Celiac disease which is a highly prevalent autoimmune mediated gastrointestinal condition occurs in 1-16% of T1DM patients compared to 0.3-1% in the general population [36]. Ingestion of wheat gluten and other related proteins have been documented to be the trigger factors of this condition in genetically susceptible individuals. Due to the associated enteropathy, patients often present with failure to thrive, chronic diarrhea and anemia due to micronutrient (mainly folate, vitamin B12) malabsorption [41].

### Screening approach for vitamin B12 deficiency among patients with T2DM

Currently, there are no published guidelines advocating for routine screening for vitamin B12 deficiency among patients with T2DM. However among type 2 diabetic patients, it is clinically plausible to screen for vitamin B12 deficiency prior to initiation of metformin and later annually among elderly patients with history of long term use of metformin (≥3-4 years), use of high doses of metformin (≥2 g/day), clinically worsening diabetic distal polyneuropathy in the presence or absence of the discussed haematological abnormalities [42].

The screening approach for vitamin B12 deficiency among diabetic patients and the general population is similar. Measurement of the serum vitamin B12 concentrations should be the preliminary screening step for vitamin B12 deficiency among patients with T2DM. Concentrations <200 pg/ml are usually diagnostic of vitamin B12 deficiency while concentrations >400 pg/ml confirm absence of vitamin B12 deficiency [43].

Measurement of serum MMA or homocysteine concentrations is a more sensitive and specific approach for screening especially among type 2 diabetic patients with borderline serum vitamin B12 concentrations of 200-400 pg/ml and subtle haematological manifestations. Serum homocysteine and MMA concentrations of 5-15 μmol/l and <0.28 μmol/l are considered within the normal range respectively [42,44].

### Screening for vitamin B12 deficiency among patients with T1DM

Among patients with T1DM, there are no clear guidelines regarding screening for vitamin B12 deficiency. However, due to the high prevalence of pernicious anaemia and subsequent vitamin B12 deficiency among T1DM patients reported in most cross sectional studies, it would be pragmatic to screen at diagnosis and then later yearly for 3 years, then five yearly thereafter or in presence of any clinical indication since vitamin B12 deficiency can develop at anytime [31]. Screening should involve assessment of serum vitamin B12 levels and markers of gastric autoimmunity like PCA and AIF especially among T1DM patients with GAD-65 and thyroid peroxidase antibodies. Presence of these auto antibodies increases the propensity to developing vitamin B12 deficiency [31,45].

### Treatment of vitamin B12 deficiency among diabetic patients

Treatment of vitamin B12 deficiency does not differ regardless of the aetiology. All patients deficient of vitamin B12 should receive replacement therapy with either oral or parenteral vitamin B12 [46,47]. Both formulations have been demonstrated to induce comparable desirable haematological and neurological improvements regardless of the aetiology of the deficiency [48]. In adult patients with T2DM, intra muscular or oral vitamin B12 in doses of 1000 μg daily for a week then once every week for 4 weeks are sufficient to correct vitamin B12 deficiency [46,47].

Among young patients with T1DM and co-existing vitamin B12 deficiency, replacement therapy with daily intra muscular or oral vitamin B12 in the dose of 100μg for a week and then monthly is satisfactory. In severe cases, parenteral or oral administration of 1000 μg/day of vitamin B12 for a week, followed by the similar dose given every week for 1 month and then later monthly is advised [31].

Concomitant folate deficiency should be treated with oral folate replacement in doses of 5 mg daily for 1–4 months. Folate administration prior to correcting vitamin B12 deficiency should be avoided because it results into progression and / worsening of the associated neurological manifestations [46].

### Therapeutic benefits of vitamin B12 replacement among T2DM patients with diabetic neuropathy

Vitamin B12 deficiency and the accompanying hyperhomocysteinemia and elevated MMA levels have been documented to cause a distinct sensory polyneuropathy that closely mimics diabetic neuropathy. Worsening of diabetic neuropathy is also noted among patients with co-existing vitamin B12 deficiency [49].

Vitamin B12 replacement has been shown to cause symptomatic improvement among patients with severe diabetic neuropathy. One meta-analysis showed that if used either alone or in combination with vitamin B complex, there was a significant improvement in the somatic symptoms like pain and paraesthesias. Three included studies also noted an improvement in autonomic symptoms with use of vitamin B12 alone [50].

Similar superior positive findings of reduction in pain and paraesthesias were also noted with use of vitamin B12 as compared to nortriptyline in a randomized, single-blind clinical trial done in Iran among 100 patients with diabetic neuropathy. This study was approved by the Ethics review board of the Isfahan University of Medical Sciences, Iran [51].

### Vitamin B12 supplementation among patients with DM

There are no guidelines to address how often patients with T1DM and T2DM should be supplemented with vitamin B12. The optimal supplementation dose of vitamin B12 is also unknown. A recently published follow-up study from the United States of America showed that administration of oral vitamin B12 among type 2 DM patients on long term use of metformin was ineffective in correcting biochemical vitamin B12 deficiency^19^. The doses of vitamin B12 in the multivitamin formulations used by the study subjects in this survey were probably inadequate to correct vitamin B12 deficiency.

This stresses the need of further studies to determine the optimal vitamin B12 supplementation dose and frequency of supplementation among patients with DM. To avert vitamin B12 deficiency especially among adult type 2 diabetic patients on long term use of metformin, it is plausible to adopt a simple and cost effective supplementation approach in diabetes care. A 1000 μg dose of vitamin B12 given annually would be sufficient to replenish the body’s vitamin B12 stores among this category of patients [52].

## Conclusions

Clinical and biochemical vitamin B12 deficiency is highly prevalent among patients with both types 1 and 2 DM. Future large and well designed studies on screening for vitamin B12 deficiency, vitamin B12 supplementation and optimal supplementation dose among type 1 and type 2 diabetic patients are warranted to help guide formulation of guidelines in diabetes clinical care. Annual screening for vitamin B12 deficiency using more sensitive methods like serum homocysteine and methylmalonic acid concentrations (in clinical settings where they are accessible) and supplementation should be adopted among diabetic patients with specific risk factors of vitamin B12 deficiency.

## Competing interests

The authors declare no competing interests.

## Authors’ contributions

Both authors equally contributed to the development of the concept and manuscript, critically read and approved the final manuscript.
